# *Ruminococcus gnavus* in the gut: driver, contributor, or innocent bystander in steatotic liver disease?

**DOI:** 10.1111/febs.17327

**Published:** 2024-11-26

**Authors:** Vik Meadows, Jayson M. Antonio, Ronaldo P. Ferraris, Nan Gao

**Affiliations:** ^1^ Department of Biological Sciences, School of Arts & Sciences Rutgers University Newark NJ USA; ^2^ Department of Pharmacology, Physiology, and Neuroscience, New Jersey Medical School Rutgers University Newark NJ USA

**Keywords:** fatty liver disease, gut liver axis, *Ruminoccocus gnavus*, steatosis

## Abstract

The human gut microbiome plays a crucial role in regulating intestinal and systemic health, impacting host immune response and metabolic function. Dysbiosis of the gut microbiome is linked to various diseases, including steatotic liver diseases. Metabolic dysfunction‐associated steatotic liver disease (MASLD), a chronic liver disease characterized by excess hepatic lipid content and impaired metabolism, is the leading cause of liver disease worldwide. Among the gut microbes, *Ruminococcus gnavus* (*R. gnavus*) has garnered attention for its association with inflammatory and metabolic diseases. While *R. gnavus* abundance correlates to liver fat accumulation, further research is needed to identify a causal role or therapeutic intervention in steatotic liver disease. This review surveys our current understanding of *R. gnavus* in the development and progression of steatotic liver diseases, highlighting its potential mechanisms through metabolite secretion, and emphasizes the need for comprehensive microbiome analyses and longitudinal studies to better understand *R. gnavus'* impact on liver health. This knowledge could pave the way for targeted interventions aimed at modulating gut microbiota to treat and prevent MASLD and its comorbidities.

## Graphical abstract

*Ruminococcus gnavus* (*R. gnavus*) can degrade mucin, modify bile acids, and secrete metabolites into circulation via the portal vein to the liver for further processing and subsequent systemic circulation. *R. gnavus* is associated with metabolic syndrome and inflammatory bowel disease severity. It is suspected that *R. gnavus* may play a role in liver function, as its increased presence is associated with excessive steatosis and delayed clearance of the hepatitis virus in both clinical and preclinical studies.

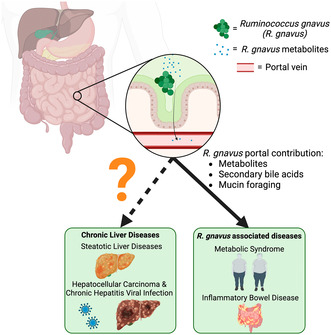

**Abbreviations**
16S rRNA: 16S ribosomal ribonucleic acid
*agg3B*
: putative invasin gene
AhR: aryl hydrocarbon receptor
CFU: colony‐forming units
*cps4J*
: capsular polysaccharide synthesis protein CpsL gene
db/db: homozygous mutation for Leptin receptor
*ermB*
: ribosomal methylase gene
*fae*D‐J: fimbria assembly genes
FDA: Food and Drug Administration, USA
HCC: hepatocellular carcinoma
IBD: inflammatory bowel disease
*kpsM*
: polysialic acid transporter gene
LEfSE: linear discriminant analysis effect size
*lnuC*
: transposon‐mediated nucleotidyltransferase gene
MASH: metabolic dysfunction‐associated steatohepatitis
MASLD: metabolic dysfunction‐associated steatotic liver disease
*mefA*
: macrolide efflux protein A gene
*msrD*
: antibiotic resistance ABC‐F gene
NAFLD: non‐alcoholic fatty liver disease
NASH: non‐alcoholic steatohepatitis
Neu5Ac: sialic acid
*papX*
: flagellum synthesis regulatory gene
*R. gnavus*
: *Ruminococcus gnavus*
*sat*
: secreted autotransporter toxin gene
*tet‐O/W/M*
: tetracycline resistance genes
TMAO: trimethylamine N‐oxide

## Introduction

The human gut microbiome, a complex ecosystem of trillions of microorganisms residing within our gastrointestinal tract, exerts a profound influence on host physiology, impacting nutrient metabolism, immune function, and hormonal signaling [1]. This intricate host‐microbiome interplay has garnered significant attention in recent years, particularly in the context of metabolic diseases.

Perturbations in the delicate balance of the gut microbiome, often referred to as dysbiosis, have been implicated in the pathogenesis of various conditions, including metabolic dysfunction‐associated steatotic liver disease (MASLD). MASLD, formerly known as NAFLD [2], represents a spectrum of chronic liver morbidities characterized by excess hepatic lipid content and impaired lipid metabolism [2, 3, 4]. With a global rise of sedentary lifestyle and consumption of obesogenic diets, MASLD has become the most prevalent chronic liver disease worldwide, poised to become the leading cause for liver transplantation in the United States by 2030 [5, 6]. Given the significant health burden associated with MASLD, understanding the factors contributing to its development and progression is of paramount importance.

**Fig. 1:**
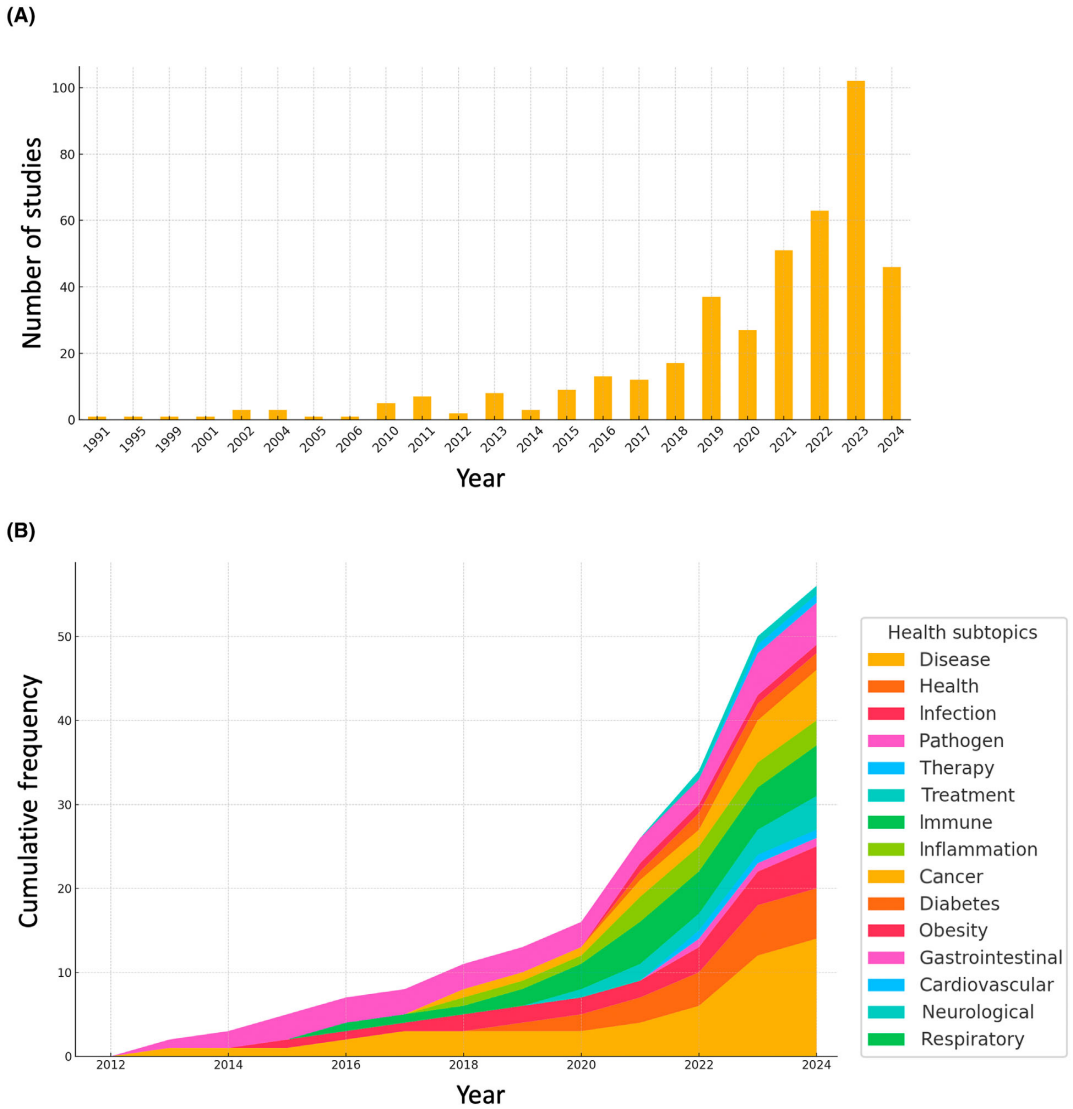
Trends in research publications on and cumulative health subtopics trends linked to *Ruminococcus gnavus*. (A) The number of research publications related to *Ruminococcus gnavus* indexed in PubMed from 1991 to 2024. From 2011 to 2015, there was a gradual increase in the number of publications, indicating a growing interest in *Ruminococcus gnavus*. This trend accelerated significantly from 2016 to the present, with a marked rise in research activity. The number of publications peaked in 2023, reflecting heightened scientific interest, possibly driven by new findings or advancements in technology that facilitate research on *Ruminococcus gnavus*. This increasing trend underscores the expanding recognition of *Ruminococcus gnavus* in scientific research, particularly regarding its role in human health and disease. (B) Research on *Ruminococcus gnavus* has surged since 2018, highlighting its growing recognition as a key player in various health contexts. This surge is particularly evident in publications related to gastrointestinal issues and infections, underscoring the bacteria's relevance to gut health and pathogenic infections. Furthermore, research has expanded to encompass a broader range of health areas, including disease, inflammation, cancer, diabetes, obesity, and cardiovascular, neurological, and respiratory conditions. This diversification reflects a growing appreciation for the complex roles *R. gnavus* may play in both health and disease. The increased focus on *R. gnavus* in gastrointestinal health aligns with the expanding understanding of the gut microbiome's influence on overall health, while the growing body of research on its role in inflammation and immune responses suggests potential implications for conditions like inflammatory bowel disease.

Among the diverse microbial inhabitants of the human gut, *Ruminococcus gnavus* (*R. gnavus*) has emerged as a commensal bacterium of particular interest, captivating researchers due to its complex and often paradoxical relationship with host health [7, 8]. *R. gnavus* has been implicated in both beneficial and detrimental effects on host physiology, and its precise role in the development and progression of MASLD remains an area of active investigation [9, 10, 11, 12, 13]. This duality has fueled a surge in research over recent years, as evidenced by the increasing number of publications focusing on *R. gnavus* and its implications for human health, particularly in areas like infection, therapy, and disease progression (Fig. 1A,B). We invite readers interested in understanding *R. gnavus'* impact on host physiology to read the most recent and in‐depth review on seminal and current studies of *R. gnavus* for a thorough introduction to *R. gnavus'* impact on health and disease [14]. In this review, this review will specifically focus on the identified and suspected contributions of *R. gnavus* to the pathogenesis of MASLD [2] and their co‐morbidities, including metabolic diseases (obesity, diabetes) and end‐stage liver diseases (liver cancer and cirrhosis).

## *R. gnavus*: a commensal with a complex identity and function

*Ruminococcus gnavus* is a core member of the human microbiota that is frequently detected in the gastrointestinal contents of humans [15]. *R. gnavus* is considered a prevalent member of the human gut microbiome, comprising between 0.1% and 2% of the gut microbiota abundance in a healthy individual [11, 16, 17]. However, variability in sampling depth of patient stool microbiome sequencing affects the accurate determination of its true prevalence in a healthy population [17]. Adding to the complexity of studying *R. gnavus* is its convoluted taxonomic history. Initially classified in the *Ruminococcus* genus, *R. gnavus* was reclassified into the *Lachnospiraceae* family, then transitioned to the *Blautia* genus, and recent categorization within the *Mediterraneibacter* genus [18, 19]. The creation of the *Mediterraneibacter* genus resulted from the classification of bacteria that are gram‐positive, non‐motile, coccus or coccobacillus, asporogenic, catalase‐positive, and obligate anaerobes [19]. This taxonomic uncertainty, coupled with the frequent grouping of *R. gnavus* with *Ruminococcus* in genus‐level analyses, presents challenges for accurately interpreting its specific contributions to host physiology [20].

Further complicating our understanding of *R. gnavus* is the inter‐strain functional diversity. Up to 74% of the *R. gnavus* genome exhibits variability across strains, leading to a wide range of functional capabilities and potentially explaining the contrasting observations regarding its role in host health [11, 21]. One notable example is the strain‐specific nature of mucolytic activity, a key characteristic linked to gut colonization. While some strains exhibit robust mucin degradation capabilities, others do not, highlighting the functional diversity within this species [9, 10]. This finding suggests that the ability to degrade mucin, a crucial aspect of gut colonization, is not universal among *R. gnavus* strains. While generally considered a commensal, accumulating evidence suggests a potential role for *R. gnavus* in inflammatory bowel disease (IBD). Studies have consistently reported increased abundance of *R. gnavus* in IBD patients compared to healthy controls. In three separate regional cohorts in the United States, *R. gnavus* abundance was found to increase in IBD patients compared to healthy controls, with an IBD‐specific clade containing 12 strains especially adapted to the oxidative stress found in the inflamed gut [11]. The ability of *R. gnavus* to combat oxidative stress and perform unique mucin foraging for self‐preservation indicates a unique adaptation of *R. gnavus* to the luminal environment and provides a competitive advantage against other bacteria to colonize and expand in the gut with the potential to affect host physiology [9, 10]. Further, *R. gnavus* virulence is suspected to be linked to its capability of adapting to the disrupted luminal environment, including oxygen tolerance, iron acquisition, and oxidative stress response [11].

## *R. gnavus*: A double‐edged sword in liver health and metabolic syndrome

The liver is responsible for filtering, metabolizing, and responding to microbial metabolites arriving via the portal vein [22]. Dysbiosis of the gut microbiome or disruption of gut barrier integrity can lead to increased circulation of microbial metabolites and pathogen‐associated molecular patterns that affect liver function through increased inflammation, lipid deposition, and fibrosis development [23]. The bidirectional communication between the intestine/intestinal microbiome and liver, termed the gut–liver axis, plays an important role in regulating liver and intestinal health. To the best of our knowledge, there is no direct evidence indicating *R. gnavus* is a causal agent in liver disease development. However, its increased abundance may impact host metabolism and microbiota composition, potentially contributing to liver disease. Studies have shown that *R. gnavus* abundance is associated with a heightened risk of liver cancer and metabolic syndrome [24, 25].

Genome‐wide association studies discovered that *R. gnavus* was one of six genera significantly associated with liver cancer outcome among Europeans and one of five enriched in liver cancer patients in Thailand [24, 26], suggesting a link between this bacterium and disease susceptibility. Similarly, patients with chronic Hepatitis B infection with immune tolerance present with increased fecal *R. gnavus* abundance, with a negative correlation with mucolytic bacterium *Akkermansia municiphila* [27]. Delayed viral clearance was detected in patients with elevated fecal *R. gnavus* and in murine models of hepatitis B infection gavaged with *R. gnavus* [27]. Interestingly, children included in this study aged 1–12 years old had a high prevalence of *R. gnavus* supporting previous findings of *R. gnavus* as an age‐discrimatory taxa [28]. *R. gnavus* abundance is also found to be enriched in fecal samples from hepatocellular carcinoma (HCC) patients compared to healthy controls, with non‐viral HCC patients presenting with greater abundance of *R. gnavus* compared to Hepatitis B‐ or Hepatitis C‐associated HCC [26]. Serum levels of intestinal fatty acid‐binding protein were elevated in HCC patients compared to healthy controls, indicating increased intestinal permeability and subsequent enhancement of gut and microbial products circulating to the liver [26]. Adding another layer of complexity, one study proposed *R. gnavus* as a biomarker for viral‐HCC tumors due to its absence in both non‐tumor tissue from viral HCC patients and in tumors from non‐viral HCC patients [29]. This intriguing finding, while requiring further investigation, highlights the potential for *R. gnavus* to differentially influence tumor development depending on the underlying etiology. The mechanism behind tumor infiltration of *R. gnavus* in viral HCC but not non‐viral HCC remains to be investigated. In this study, the identified viral HCC‐associated bacteria belonged to the *Lachnoclostridium* genus and shared 97% sequence homology with *R. gnavus*, indicating to the authors that this bacterium is indeed *R. gnavus*. These findings demonstrate the importance of deep sequencing methods in the detection of rare bacteria in fecal or tumor tissue sampling for complete bacterial identification.

MASLD encompasses hepatic steatosis with concurrent obesity, diabetes, or metabolic dysfunction [30]. *R. gnavus* has been widely implicated in metabolic diseases, like obesity and diabetes, leading to our interest in its role in fatty liver disease development. Its abundance is associated with increased adiposity, including body fat mass, body mass index, waist circumference, and serum triglycerides in men and women independent of age [25]. Further, visceral fat, pernicious adiposity, liver fat, and dietary inflammatory index score are all positively associated with *R. gnavus* [31, 32]. *R. gnavus* is also reported as an indicator of poor health due to its association with increased visceral fat and body mass index [33] and is significantly enriched in overweight and obese patients [34]. Males with metabolic syndrome and elevated γ‐glutamyl transpeptidase present with elevated fecal *R. gnavus* abundance [35]. Additionally, in male patients with elevated liver transaminases, *R. gnavus* correlates with markers of liver injury, including alanine aminotransferase, aspartate aminotransferase, and γ‐glutamyl transferase, particularly in conjunction with alcohol consumption [36].

While these studies highlight potential detrimental roles for *R. gnavus* on metabolic health, others indicate a more nuanced relationship dependent on context. For instance, supplementation of dietary fiber inulin in db/db mice, a model of obesity and type II diabetes, restores *R. gnavus* levels while simultaneously improving glucose sensitivity and beneficial microbial metabolite circulation [37], indicating a potentially beneficial role under specific conditions.

Dietary interventions further underscore the complexity of R. gnavus's role in metabolic health. Consumption of polyphenol‐rich fruit, either alone or as part of a Mediterranean diet, has been shown to reduce *R. gnavus* abundance while improving markers of metabolic health such as hepatic insulin resistance and serum low‐density lipoprotein [38]. These dietary interventions also increase the abundance of *Eubacterium eligens*, a bacterium shown to associate with a healthy diet and intestinal homeostasis [38]. A longitudinal study on diet and stool analysis found that diet quality has an inverse relationship with *R. gnavus*, *Collinsella*, *Parabacteroides*, *Ruminiclostridium_5*, and *Tyzzerella* abundance, meaning that poor diet can alter microbiota composition with deleterious health consequences [39]. The authors found that the quality of a past diet is as predictive as a concurrent diet in microbiota composition, demonstrating the long‐term influence of diet on the gut microbiota composition [39]. Collectively, these studies highlight *R. gnavus* as a bacterium of interest in metabolic syndrome and obesity. However, the mechanism behind *R. gnavus* blooms or its causal relationship with metabolic syndrome have yet to be defined.

## *R. gnavus* and steatotic liver diseases

While it has been found that only a subset of MASLD patients develop leaky gut [40, 41], it is proposed that intestinal epithelial and vascular barrier disruption are required for fatty liver development in murine models of high‐fat diet feeding [41]. The exact cause of MASLD is widely thought to result from “multiple hits” involving complex interactions among genetic susceptibilities and environmental risk factors. There are no FDA‐approved treatments for MASLD, but research into the intestinal influence of steatotic liver development aims to address this unmet need [42].

Early‐stage MASLD initiates with macrovesicular steatosis, medium‐ to large‐sized lipid droplets within hepatocytes around the pericentral zone with no accompanying inflammation. As the liver is exposed to continuous ‘hits’, whether by exogenous dietary components or endogenous signaling cues, inflammation ensues with enlarged hepatocytes containing irregularly distributed microvesicular lipid vesicles and numerous smaller lipid droplets within hepatocytes. Metabolic dysfunction‐associated steatohepatitis (MASH, formerly called NASH [2]) results from the development of microvesicular steatosis within hepatocytes and is associated with pathological mitochondrial dysfunction, hepatic inflammation, and extensive liver damage. Throughout this review, we refer to studies on NAFLD and NASH with the newly established nomenclature MAFLD and MASH, respectively.

In the past decade, the number of studies of the gut microbiome in MASLD and MASH has grown exponentially. Comparisons between obese and MASH fecal microbiomes found decreased abundance of *Ruminococcus* (*Lachnospiraceae* family) in MASH patients compared to healthy individuals and those with obesity [43]. Limitations of this study include the focused analysis of only phylum, family, and genus levels of identification of bacteria and inclusion of obese patients with liver steatosis in the obese group. Separation of obese patients with steatosis from those with no liver steatosis would strengthen our understanding of microbiota composition and its effects on obesity and fatty liver development. In contrast to findings reported by [43], positive associations between *R. gnavus* abundance and liver fat/steatosis have been established in large cohort studies [31, 44]. Further, it has been recently found that fecal levels of *R. gnavus* are increased in MASH patients compared to healthy controls [45]. Utilization of linear discriminant analysis on V3V4 16S rRNA amplicon sequencing data showed that *R. gnavus* was among two other bacterial species enriched in fecal samples from MASH patients and capable of ethanol production [45]. Deep sequencing analysis and focused investigation into specific bacterial species and products have allowed us to identify potential species for study, including *R. gnavus*.

Through shotgun metagenomic sequencing of fecal samples, *R. gnavus* has been identified as one of five most enriched bacterial species in individuals with MASLD‐cirrhosis [46] and MASLD‐HCC comorbidities, with MASLD‐HCC displaying the greatest abundance [46]. Further, *R. gnavus* is negatively correlated with cytotoxic CD8^+^ T cells in MASLD‐HCC and MASLD‐cirrhosis patients compared to healthy controls [46]. Patients with MASLD and coronary heart disease co‐morbidity present with decreased *R. gnavus* abundance compared to individuals with coronary heart disease alone [47]. Despite the importance of these studies, neither publication used a MASLD control group in their analyses, which dampens our understanding of the MASLD gut microbiome. It has been shown that increased *R. gnavus* abundance is significantly increased in microbiota samples of MASLD‐cirrhosis patients compared to healthy controls [48] and MASLD controls [49], insinuating that *R. gnavus* abundance may be involved in MASLD progression into MASLD‐cirrhosis.

In a cross‐sectional study of 1355 adults, *R. gnavus* and *Ruminococcus gauvreauii* are independently associated with hepatic steatosis prevalence in both healthy and MASLD patients [44]. This study found no sex differences nor interaction between genera and liver steatosis, meaning that there may be no causal relationship between microbiota composition and liver steatosis in the human population sampled. Interestingly, *R. gnavus* abundance significantly correlates with serum glycoprotein acetyl, a protein marker used for detection of acute‐phase inflammation and diabetes [44]. Further, *R. gnavus* is one of many bacteria (across different families, genera, and species) with a positive association to liver steatosis when energy‐adjusted diet inflammatory potential score was considered [31]. *R. gnavus* abundance, along with accompanying findings from studies in this review, are highlighted in Table 1. Together, these studies illuminate the complex and intricate relationship between the gut microbiome and liver health, a relationship we are still trying to define.

**Table 1:** Overview of *R. gnavus* abundance in metabolic disease.

*R. gnavus* relative abundance	Study findings	Disease (human or mouse)	References
Increased	Positive correlation with: Body fat and visceral fat Body mass index Waist circumference Serum triglycerides Dietary inflammatory index score Pernicious adiposity	Obesity (human)	[25, 31, 32, 33]
Decreased	Exogenous inulin improved liver histology and restored glucose sensitivity	Diabetes/obesity (db/db mouse)	[37]
Increased	Positive association with hepatic insulin resistance Significant negative association with insulin secretion Polyphenol‐rich fruit alone, or in combination with mediterranean diet, reduces *R. gnavus* abundance, hepatic insulin resistance, and serum low‐density lipoprotein levels while increasing *Eubacterium eligens* abundance	Pre‐diabetes (human)	[38]
Increased	Inverse association with diet quality	N/A (human)	[39]
Decreased		MASLD with coronary heart disease co‐morbidity	[47]
Increased		MASH	[45]
Increased	Associated with higher prevalence of hepatic steatosis (along with *Ruminococcus gauvreauii* group)	Healthy and MASLD (human)	[44]
Increased	Positive correlation with alanine aminotransferase, aspartate aminotransferase, and gamma‐glutamyl transferase	Male with elevated liver transaminases and alcohol consumption (human)	[36]
Increased		Male with metabolic syndrome and elevated gamma‐glutamyl transpeptidase (human)	[35]
Increased	Increased in males compared to females	High‐fat diet (mouse)	[54]
Increased	Increased serum low‐density lipoprotein, serum total cholesterol, and liver triglyceride Liver fibroblast growth factor 21 reduced, similar findings to high‐fat diet‐fed mice	Germ‐free mice colonized with *R. gnavus* (mouse)	[38]

While an association between *R. gnavus* and liver steatosis has been identified, the directionality of this association and the mechanism of *R. gnavus'* role in steatotic liver disease development remain unclear. Male mice fed high‐fat diet for 16 weeks or colonized with *R. gnavus* daily for 30 days (oral gavage 10^8^ CFU per g mouse weight) display increased serum markers of fatty liver disease, including low‐density lipoprotein and total cholesterol and elevated liver triglyceride levels compared to uncolonized control diet‐fed mice [38]. Further, hepatic fibroblast growth factor 21 levels, a major regulator of metabolic energy utilization, were significantly reduced following high‐fat diet feeding and *R. gnavus* colonization in these mice [38]. These data demonstrate that *R. gnavus* can directly alter host energy and cholesterol homeostasis similar to high‐fat diet feeding in a murine model of fatty liver disease.

Similarly, high‐fat diet feeding increased fecal *R. gnavus* abundance from 1% to 5% relative abundance over 12 weeks in female mice, with recognition as one of 22 high‐fat diet biomarker bacteria that are highly abundant after high‐fat diet feeding [50]. Exercise decreases *R. gnavus* abundance in high‐fat diet‐fed female mice [50], which supports previous understanding of exercise‐dependent depletion of *R. gnavus* abundance in murine models [51]. In antibiotic‐treated mice, *R. gnavus* exarcebates high‐fat diet‐induced metabolic disturbances like hepatic steatosis, liver triglyceride levels, and adiposity in male mice [52]. Genetic deletion of G‐protein coupled receptor 35 (GPR35) in mice leads to excessive weight gain, glucose intolerance, and increased abundance of *R. gnavus*, indicating a relationship between downstream GPR35 signaling, metabolic syndrome, and microbiota composition [52, 53]. *R. gnavus* abundance is found to be higher in male mice compared to female mice of the same genotype, regardless of diet [54]. Surprisingly, in this study, *R. gnavus* abundance trends toward decreased in male mice following high‐fat diet compared to male control diet mice. Further, wild‐type females display a significant reduction of *R. gnavus* and *Peptococcaceae* abundance following high‐fat diet feeding compared to control diet‐fed female mice [54]. Studies that examine the causal or mechanistic relationship between fatty liver development and *R. gnavus* are limited, but examination of the literature demonstrates a growing need to understand how *R. gnavus* may impact liver function in both preclinical and clinical settings.

## Potential contribution of *R. gnavus* metabolites on the liver and steatotic liver disease

The inherent nature of gut‐liver communication allows for microbial metabolite influence of liver function, as portal circulation accounts for 70% of blood flow into the liver. *In vitro*, *R. gnavus* can ferment inulin and lactate and produce varying levels of acetic and formic acid, short‐chain fatty acids including acetate and propionate, ethanol, and 1,2‐propanediol [9, 55, 56]. *In vivo*, *R. gnavus* has been implicated as the main producer of tryptamine, indolacetate, indolacetylglycine, and trimethylamine N‐oxide (TMAO) in mice [57, 58, 59]. Further, resistance to antibiotics like tetracycline, gentamicin, vancomycin, and erythromycin has been found widespread across the genomes of *R. gnavus* strains, likely rendering their potential virulence and pathogenicity in inflammatory diseases [21]. In patients with intrahepatic cholestasis of pregnancy, *R. gnavus* and *Lachnospiraceae* FCS020 and NK4A136 groups significantly contribute to increased metabolism of hypoxanthine, a stress metabolite resulting from purine metabolism [60]. Phenylacetic acid, an established *R. gnavus* metabolite [61], is significantly associated with hepatic steatosis in non‐diabetic obese women compared to healthy controls [62]. Insight on how nutrient availability affects *R. gnavus* survival and expansion is key to understanding its effect on the intestine and extra‐intestinal tissues like the liver.

In patients with infectious diarrheal diseases, including *Clostridium difficile* infection, *R. gnavus* abundance is negatively correlated with fecal hexanoic and pentanoic acid and positively correlated with phenylalanine [63]. The changes in carbon resources likely contribute to *R. gnavus* survival in the altered gut microenvironment, as it has been shown that *R. gnavus* catabolizes phenylalanine into phenylethylamine to promote serotonin synthesis and subsequent gut motility [61]. In obese patients with simple steatosis, there are increased serum serotonin levels compared to obese patients with normal liver or MASH [64]. In a murine MASH model, portal circulation of trimethylamine leads to its oxidation into TMAO in the liver, where it reduces hepatic fibrosis [65]. TMAO, a microbial catabolite linked to inflammation in the liver, kidney, and brain, increases in the serum of *R. gnavus* monocolonized mice compared to PBS controls [58]. While this study focused on the gut‐brain axis, it is reasonable to postulate on the effects on organs, such as the liver, which receives a larger influx of microbial metabolites. *R. gnavus* colonization in germ‐free mice also leads to increased colonic tryptamine, colonic and serum indole acetate, and serum trimethylamine levels, of which tryptamine and indole acetate are products of microbial tryptophan metabolism [58, 59, 61]. Obese patients with MASLD displayed significantly increased serum tryptophan levels, while indole acetate showed a trend toward increased when compared to obese patients with normal liver histology [64]. In this study, there were no differences between obese patients with simple steatosis or MASH, indicating a metabolic signature that develops upon the onset of steatotic liver disease [64]. Tryptamine and indole acetate serve as aryl hydrocarbon receptor (AhR) ligands to suppress inflammatory cytokines, including in resident macrophages of the liver [58, 66]. While AhR activation is suspected to reduce hepatocyte lipogenesis, there is minimal information on how bacterial metabolites regulate hepatic steatosis [66]. Alternatively, tryptamine and phenylethylamine activate the trace amine‐associated receptor 1‐extracellular signal‐regulated kinase signaling axis, reducing insulin sensitivity, which is considered a “first hit” in the development of MASLD [61]. Together, these studies demonstrate a dynamic relationship between microbiota composition and metabolite production in the development and progression of liver diseases, including MASH.

Not all bacterial metabolites result from dietary factors or bacterial *de novo* synthesis. Primary bile acids, digestive surfactants synthesized in the liver through cholesterol catabolism, can be altered by microbiota to affect human health, especially in enterohepatic diseases [67]. Bacteriotoxic properties of bile acids are circumvented by microbial enzymatic intervention [68]. Decreased secondary bile acids in murine MASH liver tissue and distinct bile acid production in IBD patients demonstrate that microbial responses to host products influence disease pathogenesis [69, 70]. Like numerous bacteria, *R. gnavus* encodes the enzymes responsible for isomerization, dehydroxylation, and amino acid conjugation of bile acids like ursodeoxycholic acid (UDCA), cholic acid, and chenodeoxycholic acid [68, 70, 71]. *R. gnavus* increases the bile acids to cholesterol ratio in patients with chronic hepatitis B infection, which leads to immune tolerance and delayed viral clearing [27]. Gallstone patients present with increased *R. gnavus* abundance and elevated serum free and secondary bile acids compared to healthy controls [72]. High‐dose treatment of UDCA reduces hepatic inflammation in a murine model of MASH [73], and elevated secondary bile acids have been found in male NAFLD patients, correlating with fibrosis score [74]. Studies assessing host bile acid signaling, especially in the context of the *R. gnavus* effect on fatty liver development, are limited. Future investigations require deep sequencing, longitudinal study design, and thorough bile acid signature analysis, as bile acid profiles may be strain‐, disease‐, and abundance‐dependent.

## Closing remarks

Our limited understanding of *R. gnavus* in the context of liver health and disease is due to various factors. First, classification and nomenclature changes over the past decade have made identification of *R. gnavus* complicated, especially when considering sequencing depth and programs used in bacterial taxa identification. In a few studies discussed in this review, the use of linear discriminant analysis effect size (LEfSE) was able to detect *R. gnavus* abundance [45]. While this tool was developed to address the need to consider effect size in the analysis of separate microbial communities [75], it comes with limitations, including reduced consistency across datasets and failure to correct *P* values in analyses output [76]. Our understanding of the strain differences in bacteria like *R. gnavus* continues to grow. It is vital that we continue to explore species‐ and strain‐specific microbial analysis in preclinical and clinical settings and push for the use of multiple methods of microbiota analysis. Combined approaches, like culturomics and metagenomic sequencing, will allow us to identify MASLD/MASH‐specific microbiota and their metabolic capacities in disease progression, especially when multiple analysis methods are employed.

Second, a deficit of longitudinal studies in patients with MASLD and MASH with microbiome species‐ and strain‐level resolution and inclusion of appropriate controls continues to be a limiting factor in the field. Delay in MASLD and MASH diagnosis and minimal consideration for microbiome analysis in personalized care both play a role in this effort. Cross‐sectional, or single‐point, studies lack the sensitivity to detect transient blooms, classified as temporary increases of > 5% relative abundance of total microbiota, which does not reflect the dynamic nature of the gut microbiome in health and disease [11]. Implementation of longitudinal studies of the gut microbiome through consistent and repetitive sample collection and analysis has become vital for the detection of minimally abundant species implicated in disease development. Greater emphasis should be placed on increased longitudinal studies of microbiome composition and metabolite production through the stages of MASLD and MASH development. Having a strategic approach to microbiome analysis will allow researchers to approach microbiome changes normalized to specific individuals.

Third, increased effort in identifying the microbiota composition and metabolite contribution in ileal content rather than stool alone will increase our understanding of gut‐to‐liver communication via portal circulation. This perhaps remains as the most technically challenging and patient‐invasive approach. As biopsy confirmation of MASLD and MASH becomes less frequent due to the development of non‐invasive screening methods, the feasibility of ileal content sampling continues to dwindle. Instead, focusing on longitudinal monitoring of MASLD and MASH patients accompanied with fecal sampling will allow clinicians and researchers to understand microbiome impact and response to disease development.

**Fig. 2:**
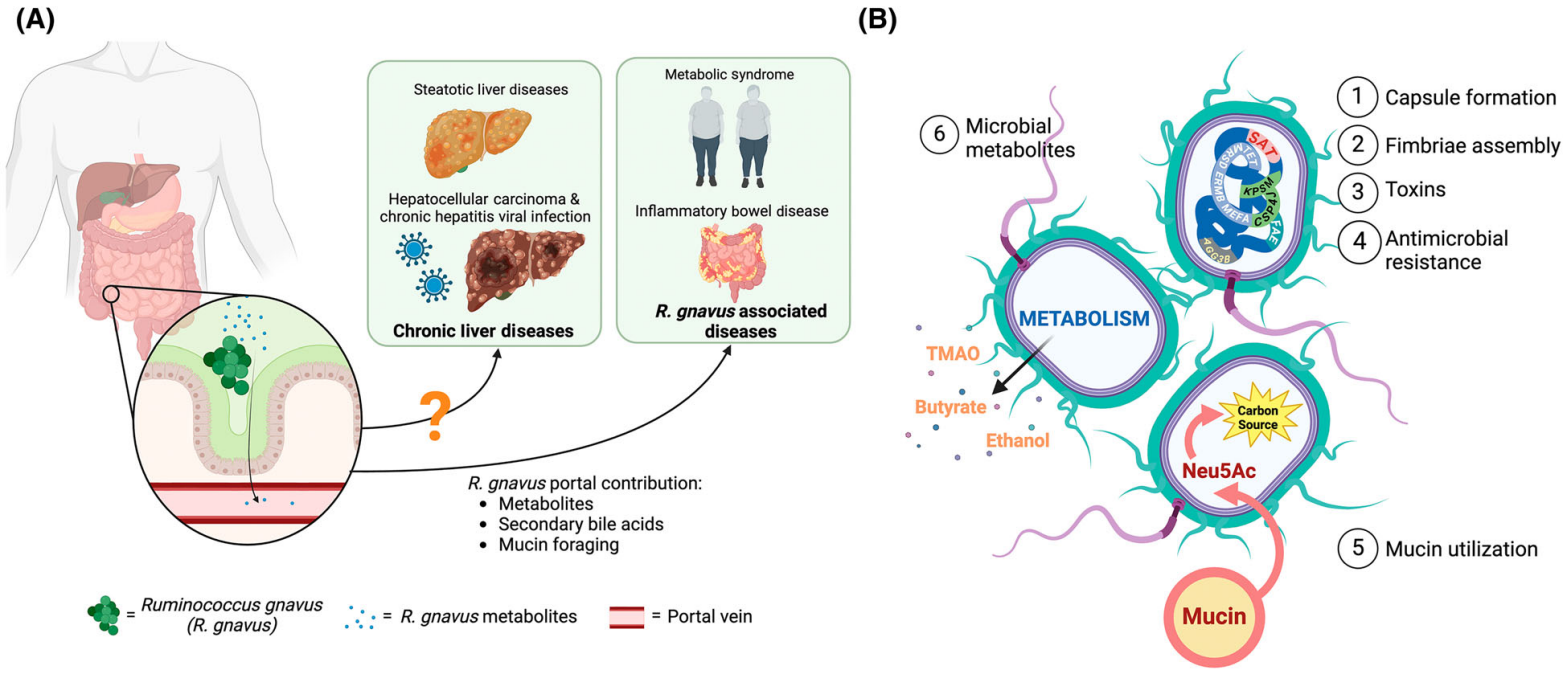
Graphical abstract and working model. (A) *Ruminococcus gnavus*, commensal bacterium in the gut, secretes metabolites and generates secondary bile acids that are circulated via the portal vein. *Ruminococcus gnavus* and its metabolites have been established in metabolic syndrome and inflammatory bowel disease, but its role in liver disease development is suspected. (B) The genetic, phenotypic, and metabolic advantages of *Ruminococcus gnavus* within the gut milieu, emphasizing its roles in toxin production, fimbriae assembly, antimicrobial resistance, mucin degradation, and microbial metabolite production. The bacterium *Ruminococcus gnavus* is depicted with its external structures, including the capsule, fimbriae, and flagella. (1) *R. gnavus* is endowed with genes involved in capsular polysaccharide synthesis, such as *kpsM* and *cps4J*, which facilitate immune evasion and colonization of host tissues. (2) It harbors fimbriae assembly genes (*faeJ*, *faeI*, *faeH*, *faeF*, *faeE*, and *faeD*) and flagellum synthesis regulatory genes (*papX*), which are critical for bacterial adherence, motility, colonization, and persistence within the gut environment. (3) Genomic analyses reveal that *R. gnavus* possesses the secreted autotransporter toxin gene *sat*, involved in adhesion to host cells, tissue invasion, and modulation of the host immune response. Additionally, the invasin protein gene *agg3B* encodes a protein that facilitates bacterial invasion of host cells, potentially enabling *R. gnavus* to replicate intracellularly and contribute to pathogenicity. (4) *R. gnavus* exhibits a repertoire of antimicrobial resistance genes, including *tetO*, *tetW*, *tetM*, and *msrD* (conferring tetracycline resistance); *lnuC* and *ermB* (conferring resistance to macrolides, lincosamides, and streptogramin B); and *mefA* (conferring macrolide resistance), endowing the bacterium with mechanisms to withstand antibiotic treatment. (5) *R. gnavus* has machinery for adhesion and mucus interaction due to its mucus‐binding properties (CBM40 domain), which are crucial for a symbiotic relationship with the gut but may contribute to pathogenicity under certain conditions. It can utilize mucin sugars through sialic acid metabolism genes (nan cluster). Its sialidases cleave sialic acid residues from mucin glycans, and using sialic acid transporters, it transports sialic acid (Neu5Ac) into the bacterial cell, where it is processed for energy or other metabolic pathways. (6) *R. gnavus* produces bioactive metabolites such as trimethylamine (TMAO), a metabolite linked to cardiovascular disease; ethanol, which at elevated levels can contribute to liver damage; and butyrate, which is generally beneficial but may have negative effects in certain contexts. Figure generated with biorender.com.

As we continue to uncover causal relationships between microbial communities, their metabolites, and host physiological response, we can better develop holistic tools for the diagnosis and prevention of disease progression. *R. gnavus* can alter the portal circulation of factors that are associated with IBD severity and metabolic syndrome (Fig. 2A). We suspect that alteration of these factors may be involved in chronic liver disease severity and outcome. Further, we highlight factors that *R. gnavus* may use to alter host physiological state, including external structures and internal secretory pathways (Fig. 2B). The increased practice of open‐access datasets on microbiota composition and metabolite analysis has allowed researchers, like us, to implement their own findings and improve our understanding of the gut–liver axis in the development of steatotic liver diseases. The functional capacity of the microbiome likely imparts a greater influence on liver function than microbiome composition changes alone, yet there is a growing need to understand the impact of individual microbiome species on hepatic steatosis and inflammation [3, 22]. It is critical to recognize the impact of unique *R. gnavus* metabolites on systemic health, as excess production of these metabolites likely orchestrates signaling pathways that impact host cell homeostatic function.

## Conflict of interest

The authors declare no conflict of interest.

## Author contributions

VM – literature search, conceptualization, original draft, figure generation, editing, and final draft; JMA – literature search, figure generation, final draft, and editing; RPF – final draft, and editing; NG –conceptualization, final draft, editing.
